# Chemistry supports the identification of gender-specific reproductive tissue in *Tyrannosaurus rex*

**DOI:** 10.1038/srep23099

**Published:** 2016-03-15

**Authors:** Mary Higby Schweitzer, Wenxia Zheng, Lindsay Zanno, Sarah Werning, Toshie Sugiyama

**Affiliations:** 1 Department of Biological Sciences, North Carolina State University, Raleigh NC 27695, USA; 2 North Carolina Museum of Natural Sciences, Raleigh NC 27601, USA; 3 Department of Anatomy, Des Moines University, Des Moines IA 50312, USA; 4 Department of Agrobiology, Niigata University, Niigata 9502181, Japan

## Abstract

Medullary bone (MB), an estrogen-dependent reproductive tissue present in extant gravid birds, is texturally, histologically and compositionally distinct from other bone types. Phylogenetic proximity led to the proposal that MB would be present in non-avian dinosaurs, and recent studies have used microscopic, morphological, and regional homologies to identify this reproductive tissue in both theropod and ornithischian dinosaurs. Here, we capitalize on the unique chemical and histological fingerprint of MB in birds to characterize, at the molecular level, MB in the non-avian theropod *Tyrannosaurus rex* (MOR 1125), and show that the retention of original molecular components in fossils allows deeper physiological and evolutionary questions to be addressed.

Medullary bone (MB) is a reproductive tissue present only in birds among living organisms^1^. It is a short-lived, rapidly deposited bony tissue arising from the endosteum (connective tissue lining the marrow space), and extending into the medullary cavity and inter-trabecular spaces of long bones of gravid female birds. MB was first identified in neognaths (2,3,4 and references therein), and later in ratites^5^. To date, it has been identified in virtually all birds, with the possible exception of some songbirds^6^.

MB is classified as a reproductive tissue because its formation and depletion are tightly regulated by serum levels of estrogens^4^,^7^ that accompany ovulation. These hormones stimulate osteoblasts within the endosteum to produce new bone that extends into and in some cases fills the medullary cavity of laying birds^8^. MB provides little structural support (but see^7^,^9^), rather its primary function is to serve as an easily mobilized source of calcium for eggshell mineralization^7^,^10^,^11^,^12^. MB is gender-specific and does not occur naturally in males or juveniles, only in mature females; however, it can be induced in male birds with the administration of excess estrogen^13^,^14^. Members of Crocodylia, which along with Aves represent the extant taxa most closely related to non-avian dinosaurs, do not produce MB, and it cannot be induced in these animals^1^,^15^,^16^.

MB is easily diagnosed in living birds, by its location (primarily long bones^4^,^7^,^9^,^10^,^17^,^18^,^19^); microstructure (highly vascular, non-lamellar collagen fibrils and isotropy, lack of secondary osteons due to extensive resorption cycles); greater mineral to collagen ratios than other bone types^10^,^18^,^19^,^20^,^21^; and stimulus (estrogen dependent^3^,^7^,^10^,^12^,^17^,^18^,^20^,^22^). Additionally, it is visible in hand sample and easily separated from overlying cortical bone (CB, Fig. 1).

It was hypothesized that MB would be present in non-avian theropod dinosaurs^23^,^24^, and thus would provide a means for unambiguous gender determination and reproductive status in extinct theropods. Because of its extensive mineralization, MB has high preservation potential, and has been identified in fossil birds^21^,^25^,^26^, including non-crown group taxa^27^. Location, texture and microstructure were recently used to diagnose MB in fossil non-avian dinosaur remains, including the theropods *Tyrannosaurus rex*^5^ and *Allosaurus fragilis*^28^ and surprisingly, two ornithischian dinosaurs not on the direct lineage to birds^28^,^29^. This distribution suggests that MB may be a basal trait for dinosaurs and, because it is not produced by crocodiles (or any living non-archosaur)^1^, may have arisen in this lineage after the divergence of Avemetatarsalia and Pseudosuchia (30 and references therein). However, because some pathologies^31^ or in some cases, metaphyseal resorption and redeposition during normal growth (e.g., compacted coarse cancellous bone^32^) may approximate microstructural aspects of MB, morphological and histological traits alone cannot provide conclusive evidence that a tissue is homologous with avian MB.

To diagnose MB in non-avian dinosaurs requires a thorough understanding of its structure, distribution, and chemical composition across modern avian taxa. Although MB contains osteoblasts (bone forming cells) like other bone, MB osteoblasts have receptors on their nuclei for estrogen^33^,^34^ making this bone type more responsive to changes in serum estrogen levels. Thus, when estrogen levels are high following ovulation, MB is rapidly deposited.

The composition and biochemistry of MB matrix is distinct from CB and trabecular bone (TB) as well^7^,^10^,^12^,^17^,^18^,^19^,^22^. ‘MB is proportionately lower in collagen, and the mineral to collagen ratio is significantly greater in medullary than cortical bone (17 and references therein). MB matrix includes more non-collagenous proteins and glycosaminoglycans than any other bone type^10^, and the level of hydroxylated matrix proteins is distinct^17^. In particular, avian MB incorporates keratan sulfate, a glycosaminoglycan not found in the matrix of other bone types^35^,^36^, where chondroitin 4-sulfate is expressed instead^37^. Some data suggest that because of these matrix differences, mineralization progresses differently in MB, resulting in smaller fibrils and a less ordered crystal emplacement, hallmarks of avian-specific reproductive bone^35^,^37^. The matrix secreted by estrogen-activated osteoblasts also contains an enzyme, tartarate-resistant acid phosphatase (TRAP) (35 and references therein). This enzyme is localized to both osteoclasts and the secreted matrix^35^.

Because MB may bear a superficial resemblance to other bone types histologically (specifically viral-induced avian osteopetrosis^31^,^38^,^39^), here we test the hypothesis that MB in non-avian dinosaurs can be chemically diagnosed using the unique structural and compositional differences outlined above. We compare non-avian dinosaur tissue with ostrich (a paleognath) and chicken (a neognath) as representatives of MB expression in the extant avian lineage. We also applied computed tomography (CT) to show that MB is organizationally distinct from cortical bone, and employ histochemical staining and immunochemistry to support a diagnosis of MB in a previously examined *Tyrannosaurus rex* (MOR 1125)^5^.

## Results

**Figure 1:**
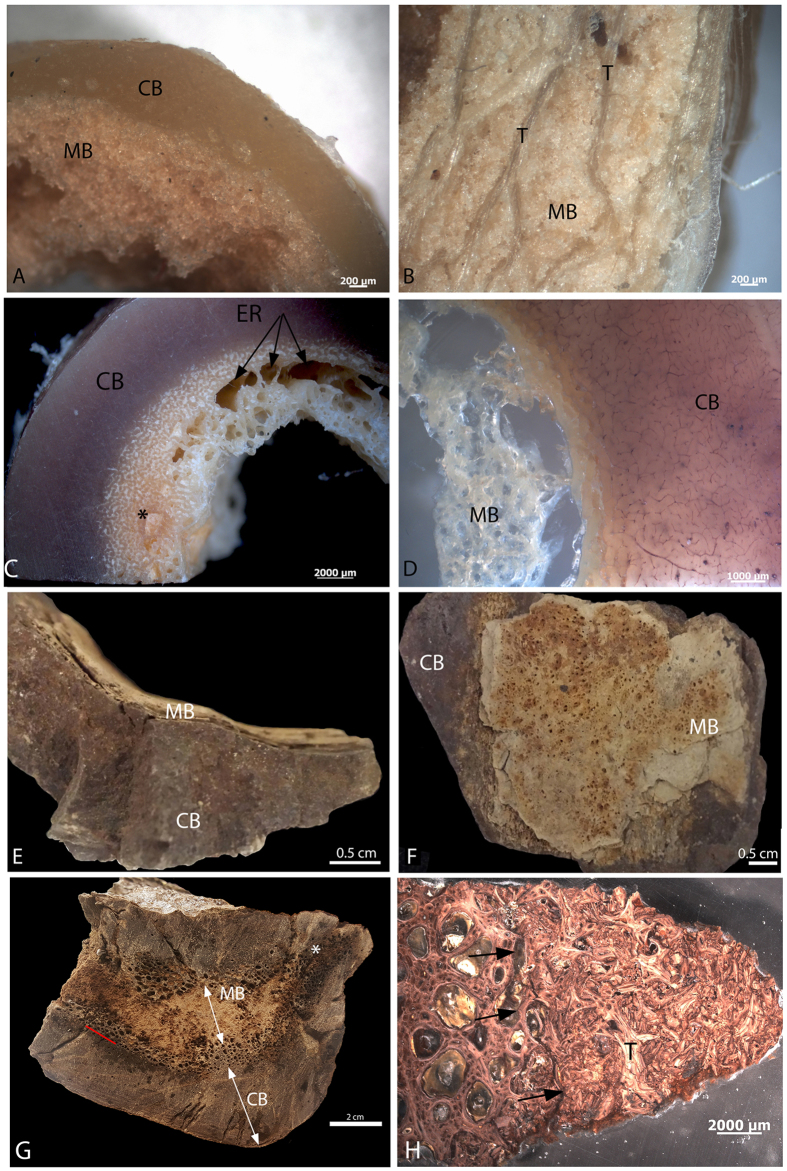
Morphological differentiation between MB and CB. (**A**) Mid shaft section from reproductively active laying hen femur shows textural differences between CB and MB. (**B**) more proximal region of hen femur shows that trabecular bone (T) can be differentiated from MB in hand sample, and that MB is deposited between trabeculae, infilling trabecular spaces. (**C**) MB in hand sample of ostrich femur appears to grade from CB, but can be differentiated by color and spiculation, as well as the presence of large erosion rooms (ER, arrows) at the boundary between layers. Infilling of erosion rooms with crystalline MB is also seen (*). (**D**) Ground section of ostrich at higher magnification shows clear separation of MB and CB. Bone fragment of MOR 1125 femur in (**E**) cross section and (**F**) medial, or medullary face orientation shows both textural and color differences between CB and MB, as well as the distinct separation between bone types. (**G**) Transverse section of MOR 1125 whole femur, showing almost complete infilling of the medullary cavity with MB. No gross deformation (corresponding to fracture callus) or bony expansion (corresponding to osteopetrosis) can be seen. Red line marks boundary between dense cortical bone and endosteal lamellar bone penetrated by multiple erosion rooms. Erosion rooms can be seen extending deep into the cortex in one region of the bone (*). (**H**) Petrographic ground section of deep CB layer and adjacent, internal MB of MOR 1125, showing change in texture and vascularity. Black arrows show distinct separation between innermost endosteal bone with erosion rooms, and region of MB deposition. Trabeculae (T) of laminar bone can be seen surrounded by MB. Scale bars as indicated.

MB is clearly visible in both extant and extinct dinosaur bone (Fig. 1), and is morphologically and texturally distinct from overlying CB and/or TB in hand sample. MB is fibrous, vascular and randomly organized, non-lamellar woven bone, consistent with its rapid deposition (e.g.^40^). In chicken (Fig. 1A,B) MB is distinct in color and texture from overlying CB (Fig. 1A) and TB (Fig. 1B). MB in ostrich femur (Fig. 1C,D) likewise shows visible differentiation in color and organization, but in hand sample, MB seems to grade from CB, becoming less dense and more spiculated as it extends toward the medullary cavity. Large erosion rooms (ER) are visible in the loosely organized MB (Fig. 1C) and at the boundary between CB and forming MB, and white crystalline MB occasionally fills the erosion rooms (Fig. 1C, *). At higher magnification in ground section (Fig. 1D), a distinct separation between ostrich CB and MB can be seen that is not obvious in hand sample. Similarly, MB is attached to, but distinct from overlying *T. rex* CB in texture, color, vascularity and organization (Fig. 1E,F). The proximal femur shaft of MOR 1125 shows no expansion or distortion consistent with osteopetrosis in gross transverse section (Fig. 1G), but cortical bone (CB) is clearly distinct from hypothesized MB (arrows), which completely fills the medullary cavity. A red line marks the distinction between CB and MB, separated by erosion rooms. These areas of porous bone can be seen to extend from the medullary cavity almost to the periosteal surface in some regions (Fig. 1G,*). A petrographic ground section is shown in Fig. 1H. Black arrows show a distinct line of separation between CB with large erosion rooms and adjacent MB, which is randomly oriented and appears fragmented. An apparent trabecular fragment (T) is seen interspersed with the MB (Fig. 1H). Figure S1 shows an expanded view of this region, with more of the inner cortical bone visible. In this microscopic section, the contrast between dense CB with secondary osteons, the region of intense resorption, and the origin of non-lamellar MB is easily visualized.

Ground sections of *T. rex* and ostrich CB is birefringent and anisotropic when observed using polarized light (Fig. S2A,C), demonstrating the lamellar nature of this bone type. MB from ostrich and *T. rex* (Fig. S2B,D) lacks birefringence, supporting the randomly oriented, non-lamellar nature of MB tissues, consistent with rapidly deposited woven bone.

**Figure 2:**
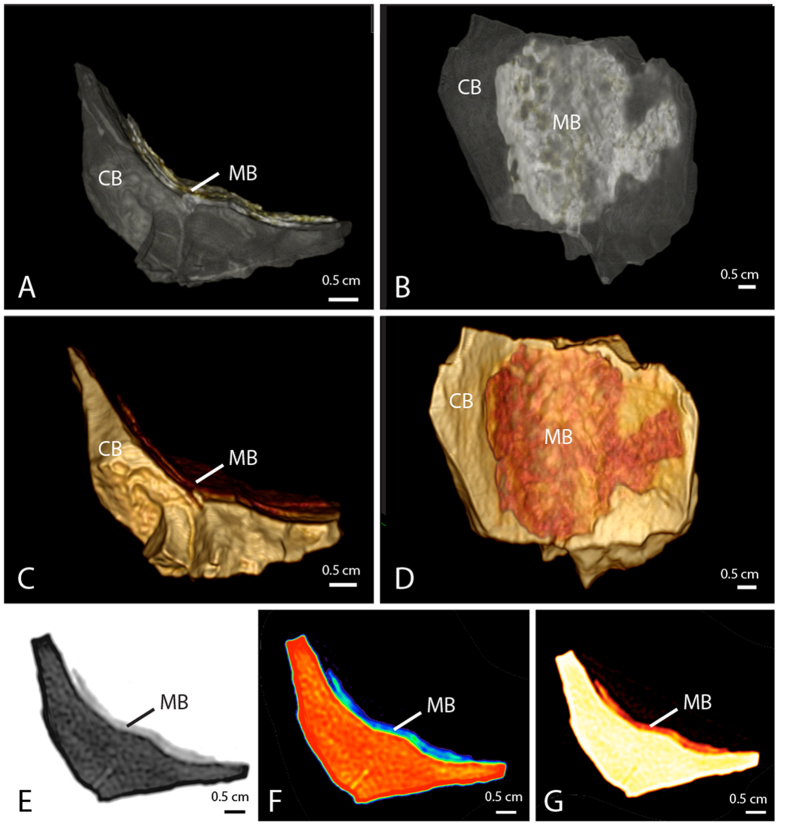
Computed tomographic imaging of MOR 1125 femur bone fragment showing morphological differentiation between MB and CB. (**A–D**) volumetric renderings and (**E–G**) cross sections. (**A,B**) High density cortical bone rendered transparent to visually isolate lower density medullary bone. (**E**) Density shown as spectrum from high (black) to low (white). Fragment is (**C,D**,**G**) color and (**F**) heat mapped. Color mapping key: (**C,D,G**) medullary bone (orange/red) and cortical bone (beige/yellow); heat mapping key (**F**): highest density (red) lowest density (blue). Sample shown in (**A,C,E–G**) cross sectional and (**B,D**) medial views.

To confirm density and organizational differences between MB and CB in this specimen of *Tyrannosaurus rex*, we employed high-resolution computed tomography (CT) (see Materials and Methods). MB in MOR 1125 is structurally unique and shows substantial density disparity with overlying cortical bone both in volumetric renderings (Fig. 2A–D) and in two-dimensional transverse section (Fig. 2E–G), independently substantiating the diagnosis of this tissue.

**Figure 3:**
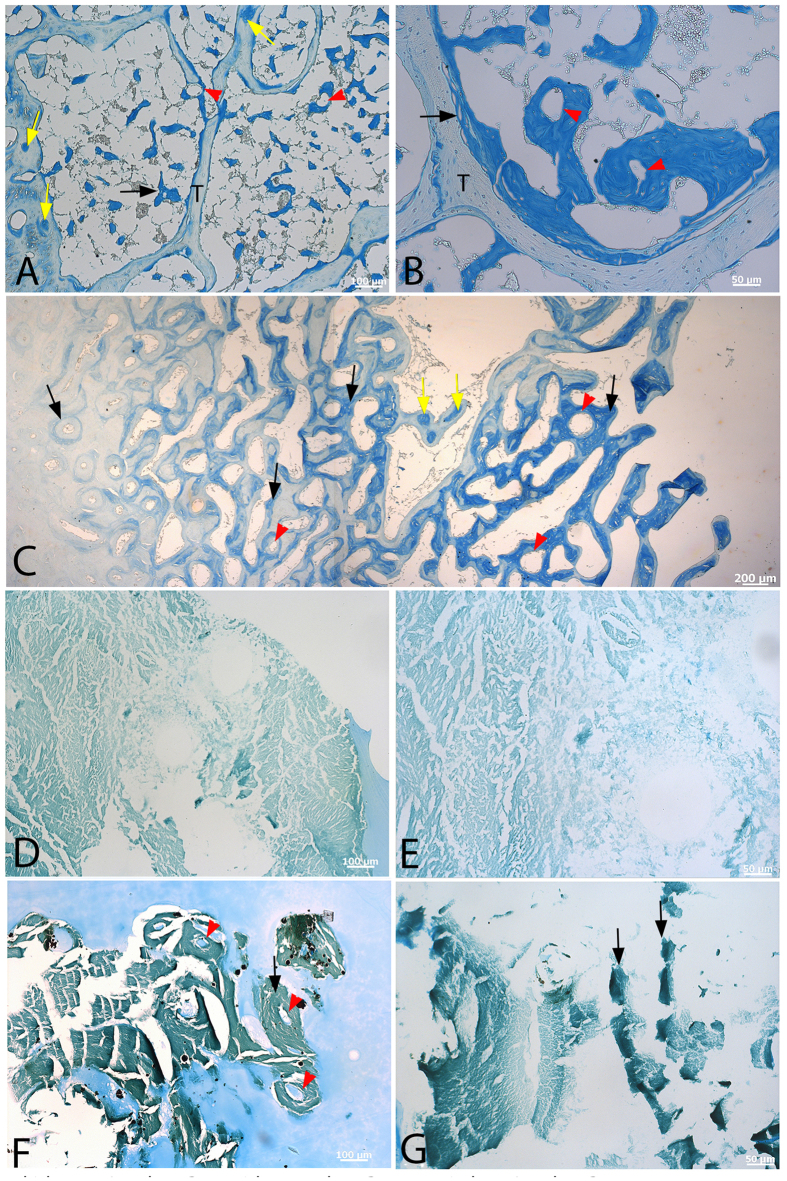
Alcian blue histochemical stain capitalizes on the differential presence of sulfated glycosaminoglycans found in MB vs CB. Low (**A**) and high (**B**) magnification of demineralized, sectioned bone from a laying hen femur show MB (black arrows), forming along the borders of CB. Alcian blue stains MB intensely, but only lightly stains CB and TB, reflecting the differences in matrix composition. MB is shown to form around ovate vacancies that may represent vessels (red arrowheads). MB is sometimes found as islands within spicules of TB or CB (yellow arrows), possibly representing centripetal infilling of pre-existing vessel channels or erosion rooms with forming MB. Ostrich (**C**), shown at a lower magnification to encompass internal-most cortical bone and developing spicules of MB. The developmental pattern differs from that of chicken, reflecting macroscopic differences seen in hand sample, where MB and CB are not distinct, but more gradational in nature. Forming MB (black arrows) can be seen lining secondary osteons (leftmost black arrow), and as pockets of bone within pre-existing cortical bone (yellow arrows). Ovate open spaces within completely formed MB spicules are also seen (red arrowheads). Low (**D**) and high (**E**) magnifications of demineralized and sectioned *T. rex* CB show fibrous matrix that is lightly stained. Dinosaur MB in low (**F**), and high (**G**) magnifications show much more intense staining (black arrows). Matrix is fibrous, but is penetrated by ovate forms within deeply staining bone (**F**, red arrows). White spaces in F are sectioning artifact, where tissue and embedding material have pulled away from bone. Scale bars as indicated.

We used Alcian blue histochemical stain (Fig. 3) to capitalize on the chemical differences between CB and MB. This stain reacts with the acidic, sulfated glycosaminoglycan keratan sulfate^35^, which is not found in cortical bone^36^,^37^. Although Alcian blue lightly stains CB in both extant (chicken, ostrich) and extinct (*T. rex*) dinosaur bone, staining of MB is much more intense in all cases, allowing microstructural and compositional differentiation of bone types. Figure 3 A,B shows low (A) and high (B) magnification images of demineralized sectioned bone from a hen in lay. Trabeculae (T) are lightly stained, supporting compositional similarity with cortical bone, but MB stains a dark blue (black arrows), and shows a pattern of MB deposition on existing trabeculae. MB arises from and lines the trabeculae and internal layers of cortical bone, but also forms as pockets within CB and TB (Fig. 3A, yellow arrows). This may be due to centripetal deposition and infilling of osteons or vessel channels with forming MB. Spicules of forming MB are occasionally penetrated by ovate “holes” that may represent vascular channels surrounded by MB matrix (red arrowheads). At higher magnifications, CB and MB are separated by large openings which may be vascular structures or, alternatively, erosion rooms that existed prior to MB deposition (Fig. 3B, black arrow).

Similarly, ostrich MB bone (Fig. 3C) reacts more intensely to the stain, supporting its distinct chemical composition, but the pattern of deposition in this ratite differs from that of laying hen. Although CB is lightly stained, similar to the chicken (Fig. 3C, left), staining intensifies with increasing depth toward the medullary cavity (black arrows), reflecting compositional differences from CB, and allowing differentiation of MB by chemistry when it is not as obvious histologically. As in the chicken, MB can be seen to form “pockets” within pre-existing CB/TB (yellow arrows) and also is penetrated by empty ovate structures (red arrowheads), supporting the possibility that MB will deposit around vascular tissues as well as pre-existing bone. Alternatively, it may be that this represents MB from a previous lay cycle that was not fully resorbed. CB from *T. rex* (Fig. 3D,E) was physically separated from underlying MB and analyzed separately (see Materials and Methods). The demineralized CB matrix is highly fibrous, and fibers show varying orientation. The matrix is only lightly stained, consistent with extant cortical bone samples, and neither the ovate structures or pockets of differentially stained regions were observed. In contrast, isolated fragments of demineralized *T. rex* MB (Fig. 3F,G) are deeply stained relative to that seen in CB, and contain regions of even more intense staining (black arrows). The MB is deposited on, or retains, empty ovate “holes”, as seen in the other MB samples (Fig. 3F, red arrowheads). The significance of these is not known, but they may represent pre-existing vascular channels on which MB is deposited.

**Figure 4:**
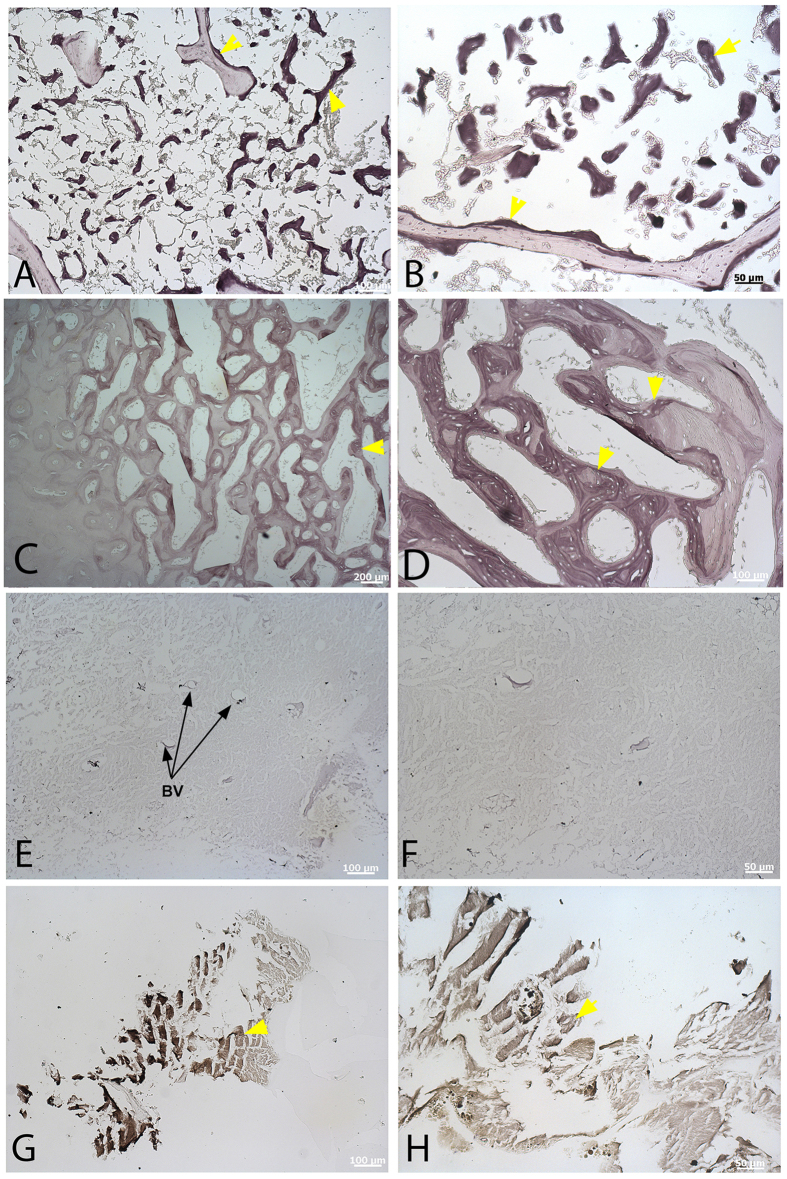
High iron diamine (HID) staining of demineralized CB and MB. (**A**) Low, and (**B**) high magnification of chicken femur showing deposition of darkly staining MB on pre-existing CB. (**C**) low, and (**D**) high magnification of ostrich femoral MB. Similar to the pattern seen using Alcian blue (Fig. 3), the distribution of MB is less distinct but can be chemically differentiated from pre-existing CB in a more mixed fashion. CB from *T. rex* femur in low (**E**) and high (**F**) magnification shows slight staining, as seen in modern samples, but staining is much more pronounced in *T. rex* MB in low (**G**) and higher (**H**) magnifications. Scale bars as indicated.

We exposed all bone types to high iron diamine (HID), a stain that reacts specifically to sulfated glycosaminoglycans^35^. All are lightly stained, but MB in extant (Fig. 4A–D, yellow arrowheads) and extinct dinosaur samples (Fig. 4G,H, yellow arrowheads) reacts much more intensely to this stain. Chicken CB and MB (Fig. 4A,B) are differentiated by intensity of staining, which reveals spicules of new bone growth along surfaces of the more lightly stained CB. Ostrich (Fig. 4C,D) shows a similar pattern. Histological distinction between CB and MB is not as clear as in chicken, with stain intensity increasing with depth of tissue into the medullary cavity; however, like Alcian blue, HID stain is capable of differentiating bone types and follows the same pattern as the Alcian blue stain. *T. rex* CB (Fig. 4E,F) is minimally stained with HID, but within the matrix, blood vessels (BV, arrows) can be seen interspersed within the fibrous matrix. The much more intense reactivity of *T. rex* MB to this stain (Fig. 4G,H) relative to CB independently supports the presence of original compounds in these dinosaur materials. Data collection parameters were identical between modern and fossil bone samples for all histochemical stains. Figure S3 shows extant and dinosaur samples, demineralized and sectioned but unstained, as controls.

This pattern is repeated when Alcian blue is combined with HID stain, a common procedure to identify MB in modern birds^35^. The dual stains (Fig. S4) differentiate MB and CB in both extant and non-avian dinosaur samples. Differential distribution of sulfated glycosaminoglycans in forming bone is clearly visualized in both chicken (Fig. S4A,B) and ostrich (Fig. S4C,D) by staining intensity; similarly, *T. rex* CB (Fig. S4E,F) shows minimal reactivity, but isolated demineralized MB sections react intensely to the dual stains (Fig. S4G,H).

**Figure 5:**
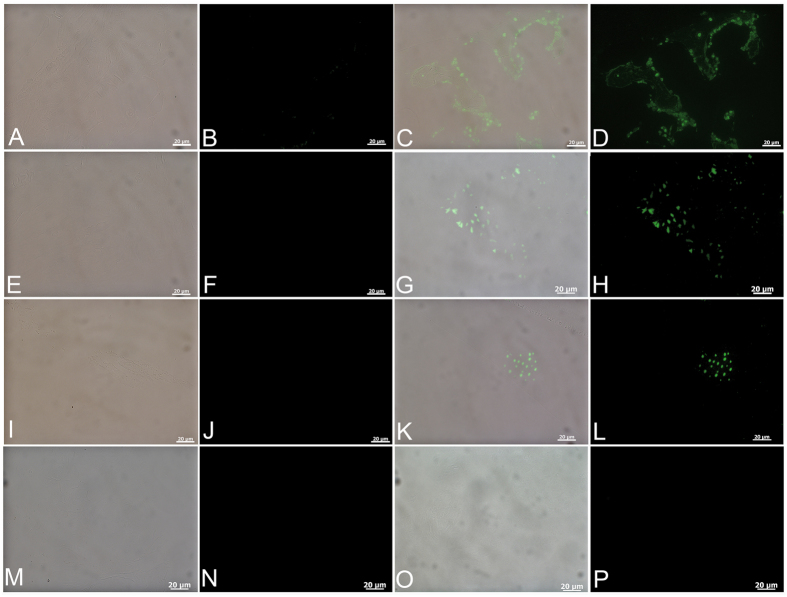
Immunochemical staining of bone using monoclonal antibodies raised against the sulfated glycosaminoglycan keratan sulfate. (**A,C,E,G,I,K,M,O**) are overlay images showing tissue and localized binding; (**B,D,F,H,J,L,N,P**) are fluorescent images using FITC label. Chicken CB (**A,B**) shows no binding; chicken MB (**C,D**) shows positive staining, with green fluorescent signal representing antibody-antigen complexes, arranged in globular clusters. Similarly, ostrich femoral CB (**E,F**) does not bind antibodies, but ostrich MB (**G,H**) is positive for binding using the same data collection parameters. *T. rex* CB (**I,J**), does not show evidence of localized antibody binding, but sections of isolated MB (**K,L**) show localized specific binding to antibodies in a globular pattern, as seen in the chicken. (**M,N**) cortical region of tarsometatarsus and (**O,P**) internal (medullary) region of chicken genetically diagnosed with avian osteopetrosis (Materials and Methods) exposed to anti-keratan sulfate antibodies. No binding is seen, using same data collection parameters.

Demineralized MB bone matrix also reacts to monoclonal antibodies specific to keratan sulfate (Fig. 5), allowing immunological differentiation of MB from CB or TB. Antibody-antigen complexes localize to globular structures within the MB matrix of Japanese quail^35^, a pattern also seen in the present study for all samples tested. No binding is visualized when antibodies are exposed to chicken cortical bone (Fig. 5A,B) but these antibodies bind MB, in a regular, globular pattern in the laying hen (Fig. 5C,D). Similarly, ostrich cortical bone is negative for binding (Fig. 5E,F), but MB shows a punctate pattern of antibody binding (Fig. 5G,H) at identical data collection parameters. *T. rex* cortical bone is negative for antibody binding (Fig. 5I,J), but a globular pattern of antibody binding is visualized in sections of demineralized dinosaur MB (Fig. 5K,L).

Avian osteopetrosis is a viral-induced pathology which results in bone deposition on the endosteal and periosteal surfaces of affected long bones, and is usually accompanied by massive expansion in bone diameter. Because this bone is rapidly deposited and endosteally derived, it may superficially resemble MB in histological section. However, bone deposition is usually bilateral^39^,^41^ and results in massive increases in bone diameter, most often accompanied by periosteal reaction and abnormality^41^. None of these features were seen grossly in our *T. rex* samples (Fig. 1G), but we applied these antibodies to sections of bone from a chicken in which DNA analyses confirmed the presence of an avian leukosis virus, the group of retroviruses that induces avian osteopetrosis^41^. Positive binding would indicate molecular similarities between the matrix of MB and osteopetrotic bone. Figure 5M,N is the cortical region of petrotic bone (Materials and Methods) and Fig. 5O,P is the internal (medullary) regions of the same bone. These controls are negative for all samples. In other control experiments, we omitted primary antibodies, but kept all other steps identical to test conditions, to control for spurious or non-specific binding of secondary antibodies or fluorescent label to the tissues (Fig. S5).

## Discussion and Conclusion

Medullary bone in extant birds is estrogen-dependent and linked to reproductive status and gender. Chemical differentiation of MB tissues in *Tyrannosaurus rex* implies that these factors can be extended deep into the theropod lineage. Homology can be inferred because of phylogenetic proximity, regional location within the skeleton, conserved histological features of endosteal derivation, high vascularity and isotropic arrangement of collagen fibers consistent with rapid deposition, as well as ephemeral nature (only 4 non-avian dinosaurs have been proposed to have this tissue), postcranial location, and now, molecular homologies. Identical tissues in multiple skeletal elements indicates a systemic process, consistent with MB deposition in extant birds, and the lack of periosteal reactive bone or abnormal bony enlargement in any elements negates an alternative hypothesis of avian osteopetrosis^39^,^42^.

The fleeting nature of MB contributes to its rarity in the fossil record, but it may be possible, through careful study, to link MB unambiguously to other, less ephemeral traits, holding potential for rigorous examination of population structure, acquisition of reproductive novelties, and ontogenetic development in non-avian dinosaurs.

Original organic components are assumed to be completely destroyed during burial and fossilization processes over millions of years. However, we have shown that tissues^43^,^44^,^45^, cells^46^ and fragments of original molecules^44^,^46^,^47^,^48^,^49^,^50^,^51^ can persist across geological time. Here we show the value of applying molecular methods to dinosaur bone to address important biological questions, using what is known of bone chemistry in homologous extant tissues.

Recently, MB was suggested to exist in pterosaurs, based upon purported histological similarity. Prondvai and Stein^52^ claimed to have identified the tissue in mandibulae of both juvenile and adult specimens, and proposed a redefinition of MB to include endosteal tissues that did not play a role in reproduction. These authors then concluded that the presence of MB could not be used for gender identification. However, the assignment of this pterosaur tissue to MB is dubious, in part because it was described *only* in mandibular symphyses, and was not identified in any postcrania examined^52^. In contrast, although MB occurs in some cranial material in a few birds, it is predominantly noted in the postcrania, specifically long bones (e.g.^3^,^9^,^10^,^19^ and references therein). It has never been reported in mandibulae of living birds, suggesting the tissue in pterosaurs is not homologous to MB. Furthermore, although MB can be induced to form in male birds with the administration of estrogen, it does not occur naturally in either males or juveniles, as was reported for pterosaurs. Because MB in living animals is estrogen dependent^14^,^34^ it cannot occur without the influence of these hormones. Thus the pterosaur tissues described by Prondvai and Stein^52^ do not meet criteria for homology with MB in extant birds or non-avian dinosaurs as described.

Here, we demonstrate that the unique chemical composition of MB in birds is retained and can be identified in a non-avian theropod dinosaur, thereby supporting the homology of these tissues with MB in living birds. We show that it is possible to remove ambiguity associated with the assignment of MB in extinct taxa, using histochemical and immunological signatures, in cases where bone tissues retain original chemical components. The application of multiple and varied molecular techniques to fossils, as well as innovations in high resolution, nano-detection instrumentation will permit the exploration of sex-linked traits in theropod dinosaurs, offering a novel approach to investigate the paleobiology of birds and their extinct dinosaurian relatives.

## Materials and Methods

Please see supplemental information for description of all specimens used in this study, as well as detailed description of methods and reagents. All procedures were conducted on bones obtained from animals after death of natural causes, or which were dead before we received them, as our experiments did not require that we use live animals. There are no university approved guidelines for experiments done on animals whose death occurred beyond the auspices of North Carolina State University; therefore no approval was required.

## Additional Information

**How to cite this article**: Schweitzer, M. H. *et al*. Chemistry supports the identification of gender-specific reproductive tissue in *Tyrannosaurus rex*. *Sci. Rep*. **6**, 23099; doi: 10.1038/srep23099 (2016).

## Supplementary Material

Supplementary Information

## References

[b1] Schweitzer M. H., Elsey R. M., Dacke C. G., Horner J. R. & Lamm E.-T. Do egg-laying crocodilian (Alligator mississippiensis) archosaurs form medullary bone? Bone 40, 1152–1158 (2007). 17223615 10.1016/j.bone.2006.10.029

[b2] Kyes P. & Potter T. S. Physiological marrow ossification in female pigeons. Anat. Rec. 60, 377–379 (1934).

[b3] Bloom M. A., Domm L. V., Nalbandov A. V. & Bloom W. Medullary bone of laying chickens. Am. J. Anat. 102, 411–452 (1958). 13617222 10.1002/aja.1001020304

[b4] Simkiss K. Calcium metabolism and avian reproduction. Biol. Rev. 36, 321–367 (1961).

[b5] Schweitzer M. H., Wittmeyer J. L. & Horner J. R. Gender-specific reproductive tissue in ratites and *Tyrannosaurus rex*. Science 308, 1456–1460 (2005). 15933198 10.1126/science.1112158

[b6] Pahl R., Winkler D. W., Graveland J. & Batterman B. W. Songbirds do not create long-term stores of calcium in their legs prior to laying: results from high-resolution radiography. Proceedings of the Royal Society, Series B 264, 239–244 (1997).

[b7] Whitehead C. C. Overview of Bone Biology in the Egg-Laying Hen. Poult. Sci. 83, 193–199 (2004). 14979569 10.1093/ps/83.2.193

[b8] Sugiyama T. & Kusuhara S. Avian calcium metabolism and bone function. Asian-Austral. J. An. Sci. 14, 82–90 (2001).

[b9] Fleming R. H., McCormack H. A., McTeir L. & Whitehead C. C. Medullary bone and humeral breaking strength in laying hens. Res. Vet. Sci. 64, 63–67 (1998). 9557808 10.1016/s0034-5288(98)90117-5

[b10] Dacke C. G. . Medullary Bone and Avian Calcium Regulation. J. Exp. Biol. 184, 63–88 (1993).

[b11] Dacke C. G., Sugiyama T. & Gay C. V. In Sturkie’s Avian Physiology, 6th Edition (ed Scanes C. G.) Ch. 25, 549–575 (Academic Press/Elsevier, 2014).

[b12] Van de Velde J. P., Vermeiden J. P. W. & Bloot A. M. Medullary bone matrix formation, mineralization,and remodeling related to the daily egg-laying cycle of Japanese quail: a histological and radiological study. Bone 6, 321–327 (1985). 3841645 10.1016/8756-3282(85)90322-9

[b13] Miller S. C. & Bowman B. M. Medullary bone osteogenesis following estrogen administration to mature male Japanese quail. Dev. Biol. 87, 52–63 (1981). 7286421 10.1016/0012-1606(81)90060-9

[b14] Ohashi T., Kusuhara S. & Ishida K. Estrogen target cells during the early stage of medullary bone osteogenesis: Immunohistochemical detection of estrogen receptors in osteogenic cells of estrogen-treated male Japanese quail. Calcif. Tissue Int. 49, 124–127 (1991). 1913292 10.1007/BF02565134

[b15] Elsey R. M. & Wink C. S. The effects of estradiol on plasma calcium and femoral bone structure in alligators (*Alligator mississippiensis*). Comp. Biochem. Physiol. 84A, 107–110 (1986). 10.1016/0300-9629(86)90050-2 2871963

[b16] Wink C. S. & Elsey R. M. Changes in femoral morphology during egg-laying in *Alligator mississippiensis*. J. Morphol. 189, 183–188 (1986). 29933649 10.1002/jmor.1051890208

[b17] Knott L. & Bailey A. J. Collagen biochemistry of avian bone: comparison of bone type and skeletal site. Br. Poult. Sci. 40, 371–379 (1999). 10475635 10.1080/00071669987476

[b18] Reynolds S. J. Mineral retention, medullary bone formation, and reproduction in the white-tailed ptarmigan (Lagopus Leucurus): A critique of Larison *et al*. (2001). The Auk 120, 224–228 (2003).

[b19] Taylor T. G. & Moore J. H. Avian medullary bone. Nature 172 (1953).

[b20] Schraer H. & Hunter S. J. The development of medullary bone: A model for osteogenesis. Comp. Biochem. Physiol. 82A, 13–17 (1985). 10.1016/0300-9629(85)90697-8 2412756

[b21] Smith N. A. & Clarke J. A. Osteological Histology of the Pan-Alcidae (Aves, Charadriiformes): Correlates of Wing-Propelled Diving and Flightlessness. Anat. Rec. 297, 188–199 (2014). 10.1002/ar.22841 24357466

[b22] Yamamoto T., Nakamura H., Tsuji T. & Hirata A. Ultracytochemical Study of Medullary Bone Calcification in Estrogen Injected Male Japanese Quail. Anat. Rec. 264, 25–31 (2001). 11505368 10.1002/ar.1101

[b23] Chinsamy A. B. & Paul M. Sex and old bones? J. Vert. Paleontol. 17, 450–450 (1997).

[b24] Martill D. M., Barker M. J. & Dacke C. G. Dinosaur nesting or preying? Nature 379, 778 (1996).

[b25] Lentaker A. & van Neer W. Bird remains from two sites on the Red Sea coast and some observation on medullary bone. Int. J. Osteoarch. 6, 488–496 (1996).

[b26] Van Neer W., Noyen K. & De Cupere B. On the use of endosteal layers and medullary bone from domestic fowl in archaeozoological studies. J. Archaeol. Sci. 29, 123–134 (2002).

[b27] Chinsamy A., Chiappe L. M., Marugan-Lobon J., Chunling G. & Fengjiao Z. Gender identification of the Mesozoic bird Confuciusornis sanctus. Nat. Comm. 4, 1–5, doi: 10.1038/ncomms2377 (2013). 23340421

[b28] Lee A. H. & Werning S. Sexual maturity in growing dinosaurs does not fit reptilian growth models. Proc. Natl. Acad. Sci. USA 105, 582–587 (2008). 18195356 10.1073/pnas.0708903105 PMC2206579

[b29] Hubner T. R. Bone Histology in Dysalotosaurus lettowvorbecki (Ornithischia: Iguanodontia)–Variation, Growth, and Implications. PLoS One 7, e29958 (2012). 22238683 10.1371/journal.pone.0029958 PMC3253128

[b30] Brusatte S. L. . The origin and early radiation of dinosaurs. Earth-Sci. Rev. 101, 68–100 (2010).

[b31] Chinsamy A. & Tumarkin-Deratzian A. Pathologic bone tissues in a turkey vulture and a nonavian dinosaur: Implications for interpreting endosteal bone and radial fibrolamellar bone in fossil dinosaurs. Anat. Rec. 292, 1478–1484 (2009). 10.1002/ar.20991 19711479

[b32] De Ricqles A. In A Cold Look at the Warm Blooded Dinosaurs Vol. AAS Selected Sympos. no. 28 (eds Thomas, R. D. K. & Olson, E. C.) 103–139 (Westview Press, 1980).

[b33] Ohashi T., Kusuhara S. & Ishida K. Immunoelectron microscopic demonstration of estrogen receptors in osteogenic cells of Japanese quail. Histochemistry 96, 41–44 (1991). 1938479 10.1007/BF00266759

[b34] Turner R. T., Bell N. H. & Gay C. V. Evidence that estrogen binding sites are present in bone cells and mediate medullary bone formation in Japanese quail Poult. Sci. 72, 728–740 (1993). 8479958 10.3382/ps.0720728

[b35] Yamamoto T. . Ultrastructrual and immunohistochemical studies of medullary bone calcification, with special reference to sulphated glycosaminoglycans. J. Electron Microsc. (Tokyo) 54, 29–34, doi: 10.1093/jmicro/dfh097 (2005). 15695482

[b36] Fisher L. W. & Schraer H. Keratan sulfate proteoglycan isolated from the estrogen-induced medullary bone in Japanese quail. Comp. Biochem. Physiol. 72B, 227–232 (1982). 10.1016/0305-0491(82)90039-6 6214368

[b37] Wang X., Ford B. C., Praul C. A. & Leach R. M. J. Characterization of the non-collagenous proteins in avian cortical and medullary bone. Comp. Biochem. Physiol. B: Biochem. Mol. Biol. 140, 665–672 (2005). 15763522 10.1016/j.cbpc.2005.01.010

[b38] Schmidt E. V., Crapo J. D., Harrelson J. M. & Smith R. E. A quantitative histological study of avian osteopetrotic bone demonstrating normal osteoclast numbers and increased osteoblastic activity. Lab. Invest. 44, 164–173 (1981). 7464041

[b39] Banes A. J. & Smith R. E. Biological characterization of avian osteopetrosis. Infect. Immun. 16, 876–884 (1977). 197009 10.1128/iai.16.3.876-884.1977 PMC421044

[b40] de Margerie E., Cubo J. & Castanet J. Bone typology and growth rate: testing and quantifying ‘Amprino’s rule’ in the mallard (*Anas platyrhynchos*). C. R. Biologies 325, 221–230 (2002). 12017770 10.1016/s1631-0691(02)01429-4

[b41] Barbosa T., Ramirez M., Hafner S., Cheng S. & Zavala G. Forensic investigation of a 1986 outbreak of osteopetrosis in commercial brown layers reveals a novel avian leukosis virus—related genome. Avian Dis. 54, 981–989 (2010). 20945777 10.1637/9138-111209-Reg.1

[b42] Simpson C. F. & Sanger V. L. A review of avian osteopetrosis: Comparisons with other bone diseases. . Clin. Orthop. Relat. Res. 58, 271–281 (1968). 4875294

[b43] Schweitzer M. H. Soft tissue preservation in terrestrial Mesozoic vertebrates. Annu. Rev. Earth Planet. Sci. 39, 187–216, doi: 10.1146/annurev-earth-040610-133502 (2011).

[b44] Schweitzer M. H. . Analyses of soft tissue from *Tyrannosaurus rex* suggest the presence of protein. Science 316, 277–280 (2007). 17431179 10.1126/science.1138709

[b45] Avci R. . Preservation of bone collagen from the late cretaceous period studied by immunological techniques and atomic force microscopy. Langmuir 21, 3584–3590 (2005). 15807605 10.1021/la047682e

[b46] Schweitzer M. H., Zheng W., Cleland T. P. & Bern M. Molecular analyses of dinosaur osteocytes support the presence of endogenous molecules. Bone 52, 414–423 (2013). 23085295 10.1016/j.bone.2012.10.010

[b47] Asara J. M., Schweitzer M. H., Phillips M. P., Freimark L. M. & Cantley L. C. Protein sequences from mastodon (Mammut americanum) and dinosaur (*Tyrannosaurus rex*) revealed by mass spectrometry. Science 316, 280–285 (2007). 17431180 10.1126/science.1137614

[b48] Organ C. L. . Molecular phylogenetics of mastodon and *Tyrannosaurus rex*. Science 320, 499 (2008). 18436782 10.1126/science.1154284

[b49] Schweitzer M. H. . Biomolecular characterization and protein sequences of the Campanian hadrosaur *Brachylophosaurus canadensis*. Science 324, 626–629 (2009). 19407199 10.1126/science.1165069

[b50] San Antonio J. D. . Dinosaur peptides suggest mechanisms of protein survival. PLoS One 6, e20381, doi: 10.1371/journal.pone.0020381 (2011). 21687667 PMC3110760

[b51] Cleland T. P. . Mass spectrometry and antibody-based characterization of blood vessels from *Brachylophosaurus canadensis*. J. Proteome Res. 14, 5252−5262, doi: 10.1021/acs.jproteome.5b00675 (2015). 26595531 PMC4768904

[b52] Prondvai E. & Stein K. H. W. Medullary bone-like tissue in the mandibular symphyses of a pterosaur suggests non-reproductive significance. Sci. Rep. 4, doi: 10.1038/srep06253 (2014).

