# Supplementary material for: Chemistry supports the identification of gender-specific reproductive tissue in Tyrannosaurus rex
Source: Sci Rep. 2016 Mar 15;6:23099. doi: 10.1038/srep23099 (PMC4791554; doi:10.1038/srep23099)
Supplement: Supplementary Information [file srep23099-s1.pdf]

Chemistry supports the identification of gender-specific reproductive tissue in *Tyrannosaurus rex*

Mary Higby Schweitzer [1,2] Wenxia Zheng [1] Lindsay Zanno[1,2] Sarah Werning[3] Toshie Sugiyama[4]

1. *Department of Biological Sciences, North Carolina State University, Raleigh NC 27695*
2. *North Carolina Museum of Natural Sciences, Raleigh NC 27601*
3. Department of Anatomy, Des Moines University, Des Moines IA 50312
4. Department of Agrobiology, Niigata University, Niigata, Japan

Materials and Methods

Materials

1. *Tyrannosaurus rex* (MOR 1125): Disarticulated partial skeleton including cranial and postcranial elements, from the base of Hell Creek Fm. near Fort Peck Reservoir, Garfield Co., MT. This specimen was determined to be ~18 years old at time of death using skeletochronological methods [1]. MB was previously described using gross and microscopic, but no chemical data [2]. Fragments of both femora and one tibia were used for previous analyses, these analyses were restricted to the left femur.

2. Ostrich (*Struthio camelus*): reproductively-active female that died with ~25 unshelled eggs in the reproductive tract (*5*). Although MB was identified in both femur and tibiotarsus, for *this* study, the proximal and midshaft regions of the femur were used. Remaining bone is housed in NCSU Molecular Paleontology labs.

3. Chicken (*Gallus gallus*), femora and tibiae from a reproductively-active female obtained from NCSU College of Veterinary Medicine, and currently housed in NCSU Molecular Paleontology labs. For these analyses, only the femur was used.

4. Chicken (*Gallus gallus*, brown leghorn), female of reproductive age (46 weeks old) with avian osteopetrosis. This specimen was obtained in 1986 from a commercial flock in Costa Rica in which ~40% of the hens were clinically diagnosed with avian osteopetrosis (based on changes in appearance, mobility, behavior, and egg production) and confirmed through post-mortem examination of skeletal lesions[3]. Later, a tarsometatarsus from one affected individual was sampled for genetic and histochemical (H&E staining) analysis [3]. DNA was isolated and amplified using avian leukosis virus (ALV)-specific primers. ALVs are the group of retroviruses known to cause avian osteopetrosis[4,5]. The presence of a novel strain of ALV similar to MAV-1 was confirmed[3]. We re-sampled this genetically-diagnosed osteopetrotic bone for additional

microanatomical, traditional staining, and immunohistochemical staining. The specimen was loaned to SW by Guillermo Zavala (corresponding author of [3]) for these and additional studies (Werning et al. in preparation).

The osteopetrotic lesion in this tarsometatarsus extends nearly the entire length of the bone, is widest at mid-diaphysis (3-4 times the normal width), and tapers towards the proximal and distal ends. This is the typical distribution of avian osteopetrosis lesions, which tend to give the leg bones a fusiform profile[6]. In cross section, the lesion is visible endosteally as well as periosteally; very little of the original metatarsal medullary cavities remain. In the deep cortex and endosteally, the lesion is compacted, but large circular and radially-oriented oval spaces are visible just under the periosteal surface around most of the circumference of the bone. This microstructural pattern is also typical of avian osteopetrosis ([3,7,8])

Methods

Computed Tomography (CT)

A femoral fragment of *Tyrannosaurus rex* (MOR 1125) (Fig. 1, [2]) was imaged at the College of Veterinary Medicine, North Carolina State University, Raleigh, NC using a Siemens SOMATOM Sensation 64 CT scanner. The fragment was scanned at 120 kV and 40 mA for 0.5 seconds (20 mA). Scanning yielded 127 slices with a slice thickness of 0.75 mm. Data were imported into Avizo (Version 8.1.0, Visualization Science Group) and OsiriX (version 5.8.2, Pixmeo SARL) for visualization, volume rendering, and image processing. Density differences were visualized under: 1) b_w inverse CLUT (Fig. 2E); 2) NIH linear table CLUT (Fig. 2F); and 3) Blackbody linear CLUT.

Ground (petrographic) sections

MB and overlying cortical bone (CB), still attached, from extant ostrich and *Tyrannosaurus rex* were embedded in Silmar 41 Clear Polyester Casting Resin (USComposites, Cat #SM-S41100). A relatively thick slice taken from Buehler isomet 1000 precision saw was attached to a glass slide with epoxy and ground to desired thickness (0.2-0.1mm)with Buehler Ecomet 4000 Grinder-polisher, and imaged with Zeiss Axioskop 40 microscope equipped with 10x objective CP-ACHROMAT 10x/0.25 (440930) for polarized light microscopy.

Histochemical Staining

1. ***Bone sample preparation***

MB and overlying cortical bone (CB), still attached, from extant ostrich and chicken femora known to be in active reproduction at time of death [2] were fixed with Neutral Buffered 10% formalin overnight, then demineralized in 500 mM EDTA (pH 8.0) until all mineral was removed, and again subjected to fixation as above. CB and MB from MOR 1125 were analyzed separately. Both bone types were demineralized in 500 mM EDTA. Because of the fragile nature of demineralized fossil bone, CB and MB from MOR 1125, taken from the region at right indicated by the red square, were embedded in 3% agar (Becton Dickinson Cat# 214530) to stabilize the tissues prior to sectioning. Extant CB and MB samples, and agar-embedded CB and MB from MOR 1125 were then subjected to routine dehydration (via Sequential incubations in 70%, 80%, 90%, 95%, and 100% alcohols for ~ 1 hour each. The tissue blocks were then placed in an additional two 100% ethanol solution to ensure that all water is removed, followed by three, 30 minute incubations in 100% xylene to clear the tissue. Tissues were then transferred to 100% paraffin, three separate, sequential incubations for 30min each to complete infiltration, then embedded in paraffin wax (Paraplast Plus EMS CAt#19216) for sectioning. Sections were taken at 5 um, using a Leica RM 2255.

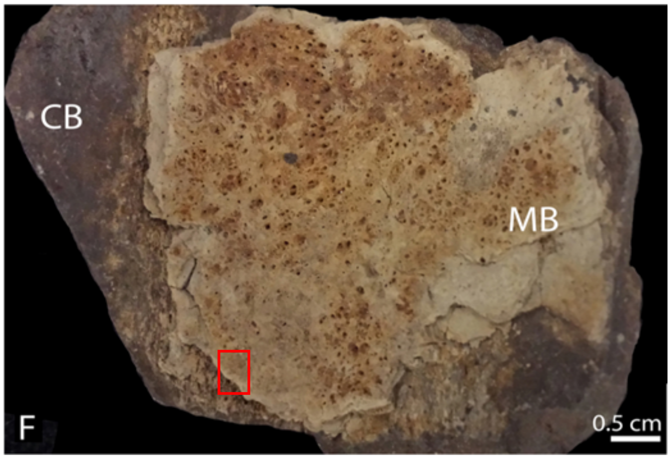

2. *Staining procedures*

Histochemical stains used to differentiate and diagnose MB in living birds were applied to both dinosaur tissues and extant controls. These stains are not conclusive, as other tissue components will react to the stains (e.g acidic polysaccharides in tissues such as cartilage); however in bone samples these can differentiate type and amount of mucopolysaccharides and glycosaminoglycans incorporated into the matrix.

A) *HID/Alcian blue stain.* Following the high iron diamine (HID) method outlined by Spicer [9], paraffin embedded sections were deparaffinized with xylene and dehydrated through a graded ethanol series. Demineralized bone sections were oxidized in 1% H_5IO_6 (periodic acid) for 10min, rinsed under running tap water, and stained with freshly made high iron diamide (HID) solution, prepared as follows: 120mg of meta diamine and 20mg of the para isomer were dissolved in 50ml of water, then poured into a glass container with 1.4ml of *N.F. 10%* ferric chloride (*$FeCl_3$*), equivalent to a 62% w/v solution of $FeCl_3.6H_2O$) for 18hr. Sections were then rinsed with water then stained in alcian blue -8GX (1% in 3% acetic acid) for 30min, rinsed again, then dehydrated with a graded ethanol series, followed by several incubations with 100% xylene. Mounting medium (Poly-Mount, PolySciences Cat#08381) and cover glass were applied for visualization.

B) *HID Stain only*. The same procedure as above was applied to a second set of bone sections, with the omission of the Alcian blue step. This demonstrates that HID components alone reacted with specificity to the differences in bone matrix chemistry.

C) *Alcian blue -8GX.* Demineralized, paraffin embedded sections of modern and ancient bone tissues were dehydrated as described above. Demineralized bone sections were oxidized in 1% H_5IO_6 (periodic acid) for 10min, rinsed under running tap water, then exposed to Alcian blue -8GX (1% in 3% acetic acid) for 30min, rinsed again, then dehydrated with a graded ethanol series, followed by several incubations with 100% xylene. Mounting medium and cover glass were applied as above.

D) *No stain control*
Sections of bone tissues were treated as above; i.e., demineralized fragments of CB and/or MB were embedded in either paraffin, or agar and paraffin. 5 μm sections were taken as described, then deparaffinized in xylene and dehydrated in a graded ethanol series. Demineralized bone sections were oxidized in 1% H_5IO_6 (periodic acid) for 10min, rinsed under running tap water, then dehydrated with a graded ethanol series, followed by several incubations with 100% xylene to clear remaining paraffin. Mounting medium was applied, and sections were imaged without further treatment.

Immunofluorescence

Demineralized *T. rex* (MOR 1125) MB, *T. rex* CB and 10% formalin-fixed demineralized ostrich and chicken MB and CB, and chicken osteopetrotic bone, were embedded in LR White resin blocks after partial dehydration in 70% ethanol and infiltration with pure LR White water permeable embedding medium (Ted Pella) as previously described (e.g. [10,11]). 200 nm sections were cut on a Leica EM UC6 Ultramicrotome, transferred to six-well, Teflon-coated slides, and dried overnight at 45°C on a warming plate.

Sections were etched with Proteinase K (PCR grade, Roche, 25 ug/ml) in 1X phosphate buffered saline (PBS) buffer at 37˚C to expose epitopes, followed by two incubations in 500 mM EDTA (pH 8.0) and two incubations in 1 mg/ml sodium borohydride for 10 minutes each for antigen retrieval (http://www.ihcworld.com/_intro/antigen-retrieval.htm). Sections were then incubated for 2 hours in normal goat serum (NGS) diluted to 4% in PBS to occupy non-specific binding sites and prevent spurious binding. Sections were then incubated with primary antibody consisting of monoclonal mouse anti-KS [Keratan Sulfate (5D4)] (Cosmo Bio Co., LTD Cat# PRPG-BC-M01), diluted 1:20 in primary dilution buffer overnight at 4°C as recommended by the manufacturer. Sections were washed multiple times to remove unbound antibody, then all sections, including controls (no primary antibodies were applied but all other steps and conditions were kept identical), were then incubated with secondary antibody (biotinylated goat anti-mouse IgG (H+L) (Vector BA-9200), diluted 1:500 in antibody dilution buffer [12] for 2 hours at room temperature. Fluorescein Avidin D (FITC, Vector Laboratories A-2001) diluted 1:1000, was applied to all sections and allowed to bind for 1hr at RT. All incubations were separated by sequential washes (2 times for 10 minutes each) in PBS w/Tween 20 (ACROS Organics) followed by two 10minute rinses in PBS. Finally, sections were mounted with Vectashield Anti-Fade mounting medium (Vector H-1000), and coverslips applied. Sections were examined with a Zeiss Axioskop 2 plus biological microscope and captured using an AxioCam MRc 5(Zeiss) with 10x ocular magnification, and data collected using the Axiovision software package (version 4.7.0.0).

Supplemental Figures:

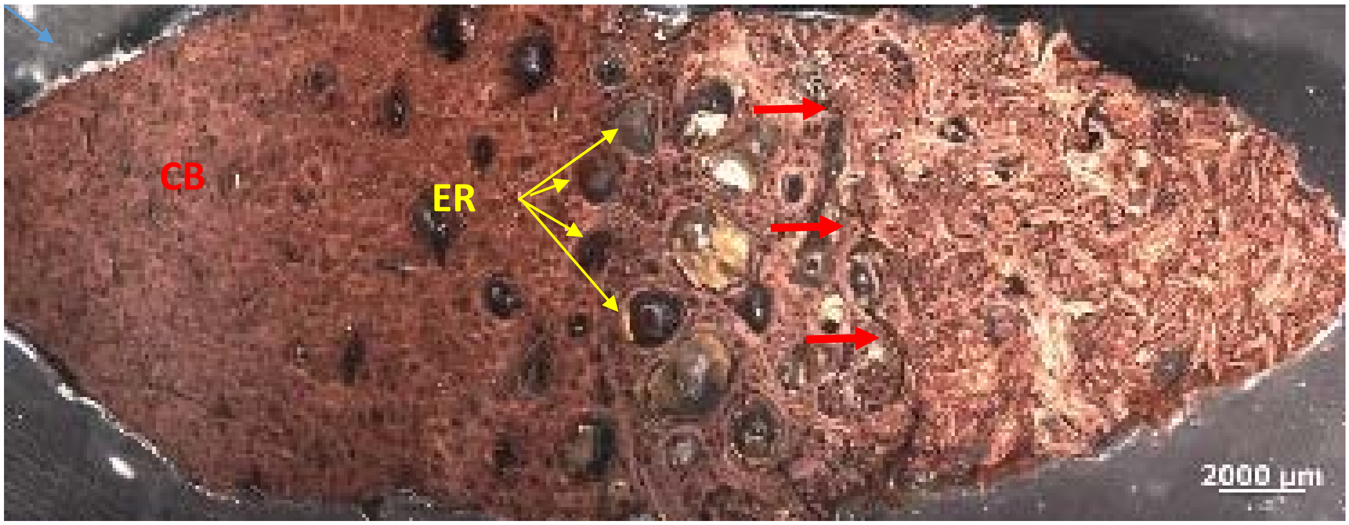

Figure S1. Complete ground section of MOR 1125 femur fragment. CB shows secondary osteons interspersed with increasingly large erosion rooms (ER, yellow arrows). Red arrows show distinct boundary between CB and non-lamellar MB.

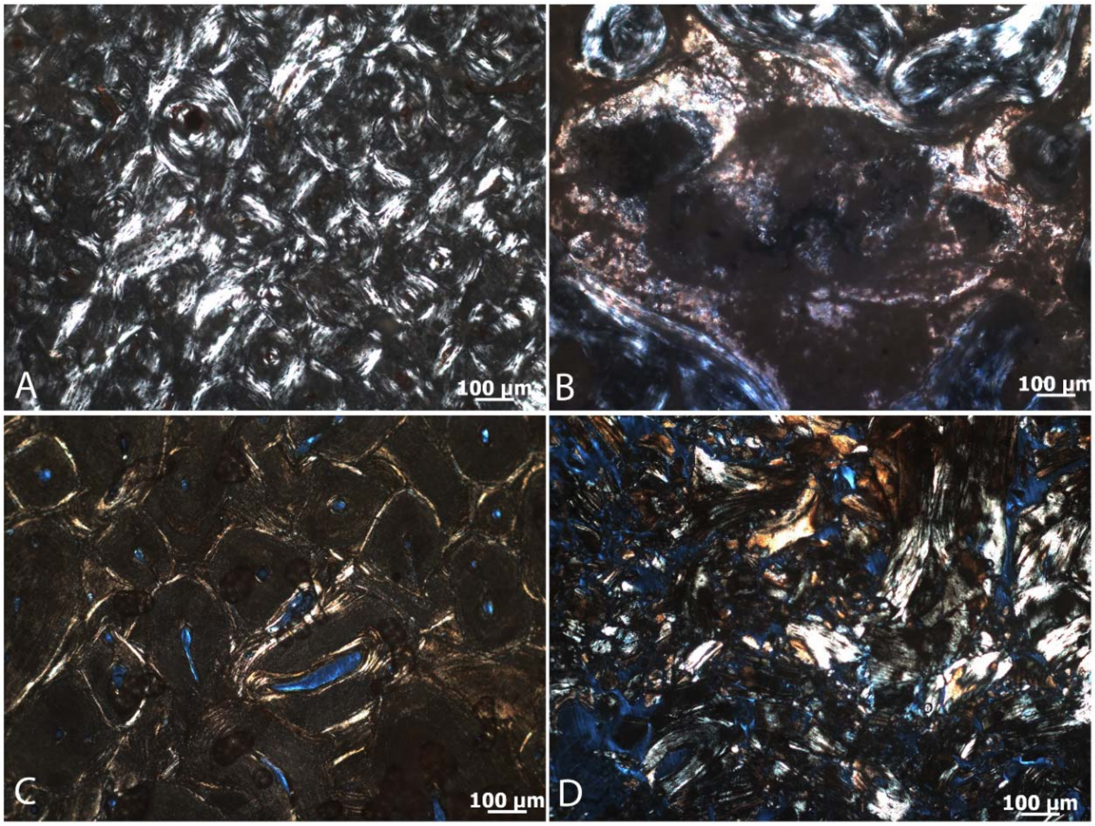

Figure S2. Petrographic sections of ostrich cortical (A) and medullary (B) bone, and *T.rex* (MOR 1125) cortical (C) and medullary (D) bone viewed in polarized light. In A and C, cortical bone demonstrates secondary osteons and birefringence, reflecting the organized lamellar nature and orientation of collagen fibers. In contrast, MB (B, D) shows non-lamellar bone with non-birefringence and isotropy, consistent with rapidly deposited, random arrangement of fibers.

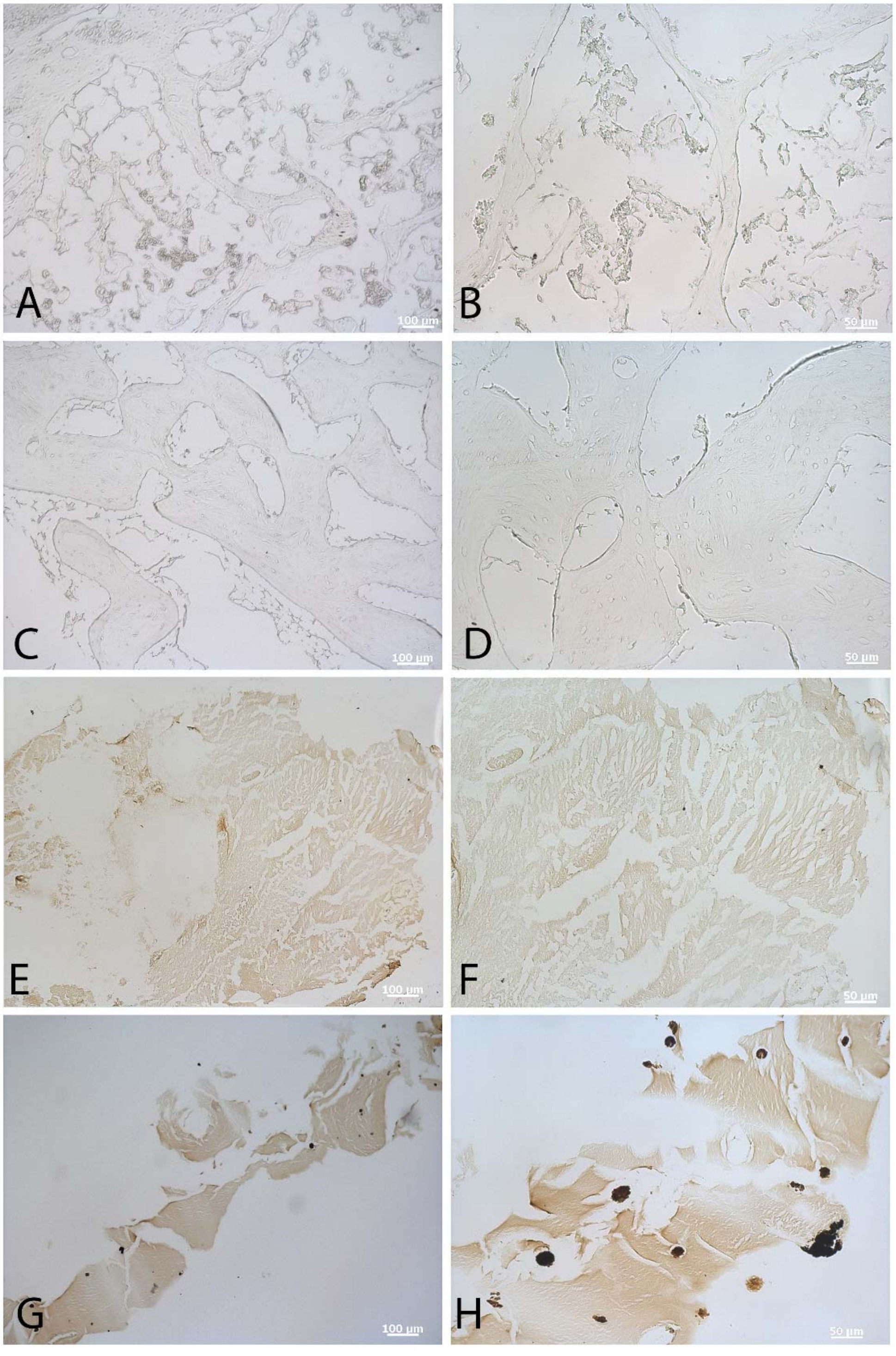

Figure S3. Unstained, untreated demineralized sections of chicken CB and MB in low (A) and higher (B) magnifications. Ostrich demineralized fragments, unstained, in low (C) and higher (D) magnification. Unstained, demineralized *T.rex* cortical bone in low (E) and higher (F) magnification show a natural brown tint to bone. Demineralized *T.rex* MB shows similar natural coloration in low (G) and higher (H) magnification.

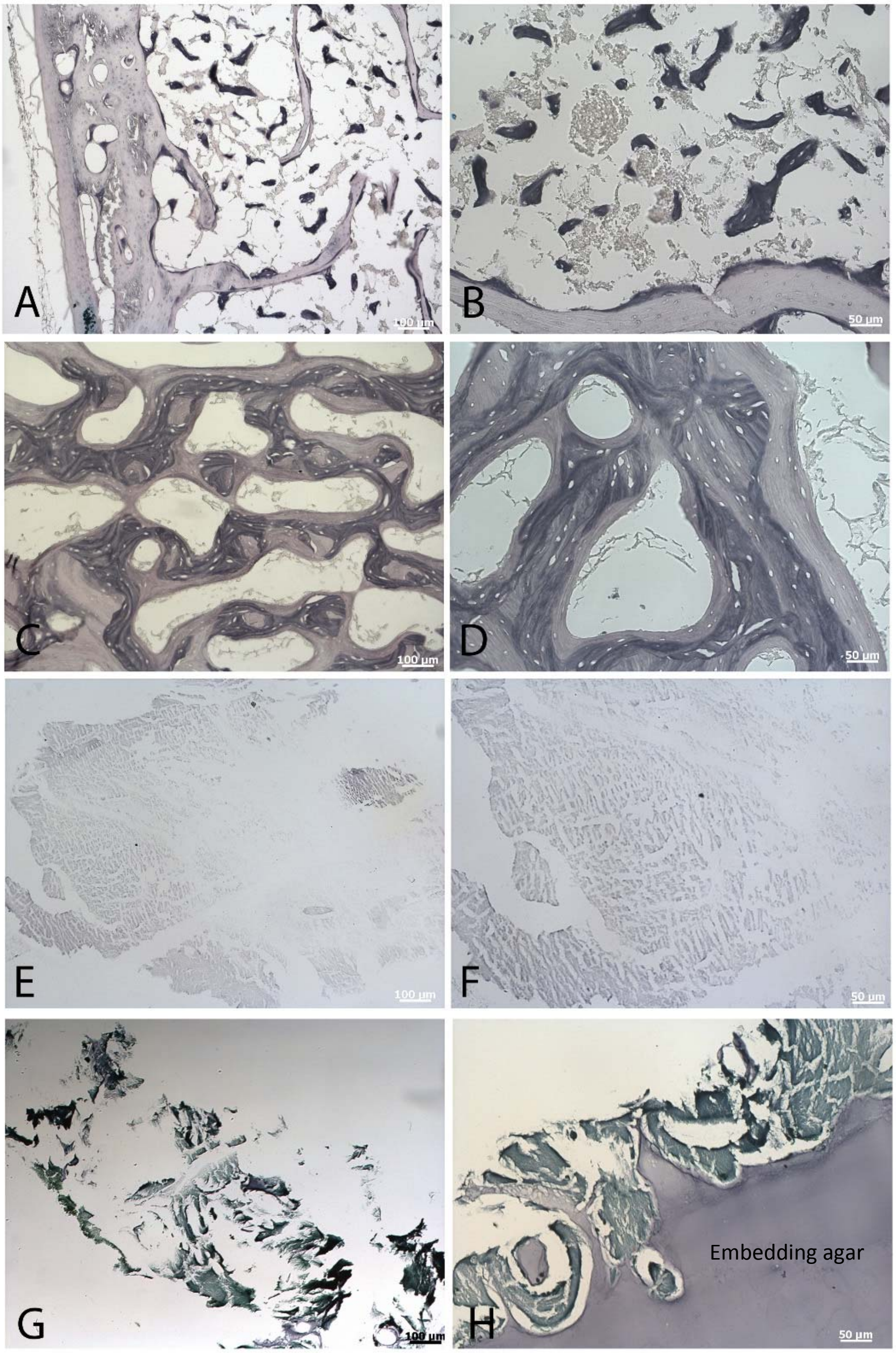

Figure S4. Demineralized chicken bone in low (A) and higher (B) magnifications, treated with both Alcian blue and HID. The dual stains demarcate CB (lightly stained) from intensely stained MB, capitalizing on chemical differences. Ostrich bone in low (C) and higher (D) magnifications shows clear differentiation of CB (lighly stained) and MB (deeply staining), showing similar chemical differentiation. Demineralized T.rex CB in low (E) and higher magnification does not react to the dual stains, while T.rex MB (G, H) is intensely stained

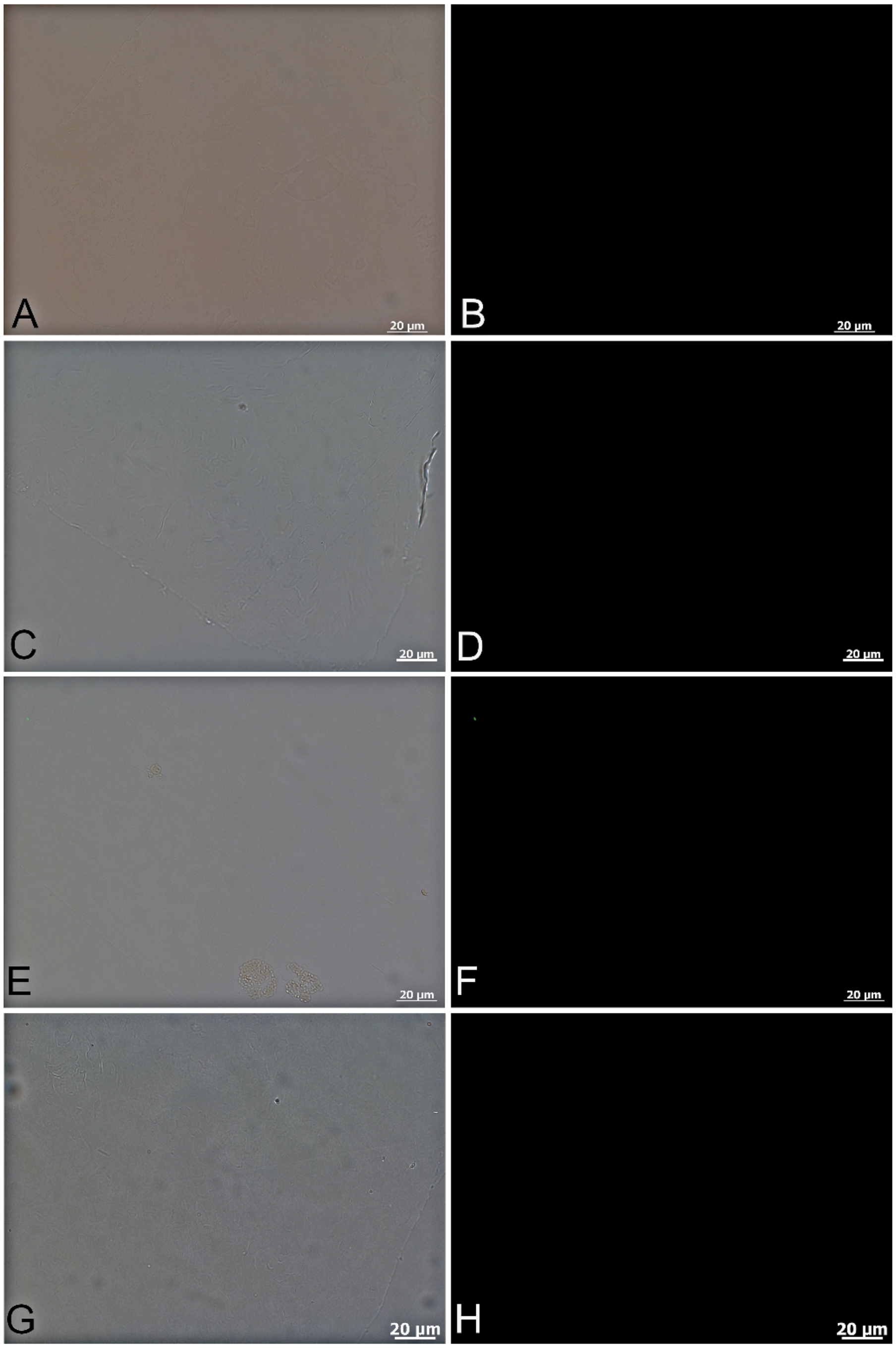

Figure S5. Controls for immunohistochemical analyses, in which no primary antibody was applied, but all other parameters, including data collection parameters, were kept identical to test conditions, to control for false positives due to spurious binding of secondary antibody. These images were taken from the same samples as presented in the text, at the same parameters; no reactivity to antibodies is demonstrated. A, C, E, G are overlay images; B, D, F, H are fluorescent images. A, B) Chicken medullary bone; C, D) Ostrich MB; E, F) Isolated MB from *T.rex* (MOR 1125); G, H) internal bone from avian osteopetrotic bone (see text).

Citations for Supplemental material.

1 Horner, J. R. & Padian, K. P. Age and growth dynamics of *Tyrannosaurus rex. Proceedings of the Royal Society, Series B* **271**, 1875-1880 (2004).

2 Schweitzer, M. H., Wittmeyer, J. L. & Horner, J. R. Gender-specific reproductive tissue in ratites and Tyrannosaurus rex. *Science* **308**, 1456-1460 (2005).

3 Barbosa, T., Ramirez, M., Hafner, S., Cheng, S. & Zavala, G. Forensic investigation of a 1986 outbreak of osteopetrosis in commercial brown layers reveals a novel avian leukosis virus—related genome. *Avian DIseases* **54**, 981-989 (2010).

4 Robinson, H. L. *et al.* 5' Avian leukosis virus sequences and osteopetrotic potential. . *Virology* **190**, 866-871 (1992).

5 Robinson, H. L., Reinsch, S. S. & Shank, P. R. Sequences near the 5' long terminal repeat of avian leukosis viruses determine the ability to induce osteopetrosis. *Journal of Virology* **59**, 45-49 (1986).

6 Banes, A. J. & Smith, R. E. Biological characterization of avian osteopetrosis. *Infection and Immunity* **16**, 876-884 (1977).

7 Powers, B. E., Norrdin, R. W., Snyder, S. P. & Smith, R. E. A sequential study of bone lesions caused by isolates of an avian osteopetrosis virus, MAV-2(0). . *Bone* **8**, 231-240 (1987).

8 Schmidt, E. V., Crapo, J. D., Harrelson, J. M. & Smith, R. E. A quantitative histological study of avian osteopetrotic bone demonstrating normal osteoclast numbers and increased osteoblastic activity. *Laboratory Investigation; a Journal of Technical Methods and Pathology* **44**, 164-173 (1981).

9 Spicer, S. S. Diamine methods for differentiating mucosubstances histochemically. *The Journal of Histochemistry and Cytochemistry* **13**, 211-234 (1965).

10 Schweitzer, M. H. *et al.* Analyses of soft tissue from Tyrannosaurus rex suggest the presence of protein. *Science* **316**, 277-280 (2007).

11 Zheng, W. & Schweitzer, M. H. in *Forensic Microscopy for Skeletal Tissues Methods and Protocols* Vol. 915 (ed Lynne S. Bell) Ch. 10, 153-172 (Methods in Molecular Biology, 2012).

12 Schweitzer, M. H., Zheng, W., Cleland, T. P. & Bern, M. Molecular analyses of dinosaur osteocytes support the presence of endogenous molecules. *Bone* **52**, 414-423 (2013).
